# Genomics and Personalized Nutrition

**DOI:** 10.3390/nu13041128

**Published:** 2021-03-30

**Authors:** Iwona Rudkowska

**Affiliations:** Endocrinology and Nephrology Unit, CHU de Québec-Laval University Research Center and Department of Kinesiology, Laval University, Québec, QC G1V 4G2, Canada; iwona.rudkowska@crchudequebec.ulaval.ca; Tel.: +1-418-525-4444 (ext. 46380)

Genomics technologies can be used to study the relationship between the human genome, nutrition, and health. For example, food components can interact with genes and their expression to alter phenotypes. Consequently, genes can influence absorption, metabolism, or transport of food nutrients or its site of action and thus influence the overall response to the diet [1]. Food compounds can also produce changes in the profile of the gut microbiome [2]. Knowledge of changes in the microbiome is valuable for an optimal diet. Furthermore, in the interaction of genes and diet, the effect of diet on individual health depends on the specific genotype [1]. Thus, genomics technologies can improve the understanding of the relevant metabolic pathways to the risk of diseases and the responses to diet. Such genomics data can facilitate the development of personalized nutrition recommendations. The goal of personalized nutrition is to prevent the onset and development of diseases by targeting dietary recommendations to an individual’s characteristics, such as genetic information, disease status, dietary intake, nutrient status, anthropometrics, physiological state, food preferences, lifestyle, and sensory preference [3]. Research findings demonstrate that some individuals may respond differently to nutrition recommendations and thus may benefit from other dietary recommendations [4]. Thus, the development of personalized nutrition should improve health and decrease the risk of disease.

The purpose of this Special Issue was to expand and add to the research which uses genomics technologies in the development of personalized nutrition recommendations. This Special Issue on “Genomics and Personalized Nutrition” features five original articles [5,6,7,8,9] and four reviews [10,11,12,13] which examine two facets of personalized nutrition: 1—genomics and food bioactive compounds; and 2—genetic variations and diet interactions.

Food bioactive compounds may modify metabolic syndrome risk factors. Metabolic syndrome risk factors include abdominal obesity, atherogenic dyslipidemia, hypertension, and hyperglycemia (insulin resistance). Additionally, inflammation may be considered as one of the metabolic syndrome components. Two articles in this Special Issue establish the impact of food bioactive compounds on several genomic parameters, such as gene expression and microbiome, to understand the effects on metabolic syndrome. First, Durand et al., 2020 [5] demonstrate that a supplement of milt herring hydrolysates improves glycemia, reduces inflammation, modulates gene expression in the liver, and alters Lactobacillus abundance. Overall, this study suggests that milt herring is a novel marine ingredient to help fight against metabolic syndrome. Secondly, Cifre et al., 2020 [6] show that the administration of all-trans retinoic acid regulates the expression of key inflammatory and lipid metabolism genes in an ex vivo system of peripheral blood mononuclear cells in normal-weight and overweight–obese subjects. These studies are excellent examples of the use of various genomics techniques to further understand the effects of foods on health and to adapt our current recommendations. 

Further, the use of multiple genomics in nutrition research requires sophisticated integration analysis methods. In the article by Khorraminezhad et al., 2020 [10], machine learning analyses are defined and nutrition studies that use machine learning techniques are examined in-depth. Overall, the review concludes that use of machine learning should complement traditional statistical analyses to fully explain the impact of nutrition on health and disease using genomics techniques [10]. 

In this Special Issue, we present three original studies and one review which examine the interaction between genetic variants and lifestyle factors, clinical features, or dietary intake. First, the article by Furukawa et al., 2020 [7] demonstrates that three genetic variants at the 12q24 locus are associated with black tea consumption in Japanese populations. Results suggest that certain individuals may drink more black tea due to their genetic profile [7]. Secondly, Jee et al., 2020 [8] show that age-related cataract risk had a positive association with polygenetic risk scores related to crystallin metabolism. Further, the genetic risk for age-related cataract development was modified by age, the presence of hypertension, hyperglycemia, sodium and coffee intake, and a Western-style diet [8]. The authors recommend that individuals with potential genotypes at risk of age-related cataracts, such as family histories, should be careful to avoid the dietary and lifestyle factors linked to age-related-cataracts [8]. Thirdly, the article by Galmés et al. 2020 [9] discusses the importance of micronutrients and the genetic variants for the immune system in the context of Coronavirus disease 2019 (COVID-19). Additionally, the study reveals that intake levels of Vitamins D, C, B12, and iron are inversely associated with higher COVID-19 incidence and/or mortality, particularly in populations genetically predisposed to lower circulating levels of these micronutrients [9]. Forth, the review by Golan et al., 2020 [11] examines how genetic variations that can affect human milk content. The authors conclude that genetic variations that can affect human breastmilk are rare but may be more common in some ethnic groups [11]. Overall, the new results presented in this Special Issue can be used to develop novel personalized nutritional recommendations based on food preference for those at risk of defects in metabolism for optimal health and prevention of disease.

Further, two review studies examine the current state of nutrigenetics recommendations and “direct-to-consumer genetic testing” (DTC-GT) services. First, Mullins et al., 2020 [12] provide examples on how genotypic information could be used to inform nutritional recommendations as well as examining ethical considerations and practical applications for using genetic information to inform dietary choices. Secondly, the review by Floris et al., 2020 [13] scrutinizes the online nutrigenetics services offered by 45 companies worldwide regarding the types of nutritional traits analyzed and the level of scientific precision of the services proposed. The authors conclude the need for specific nutrigenetics guidelines, to ensure quality standards for the nutrigenetics services offered to the customer [13]. These review studies provide an excellent update on current knowledge in gene–diet interactions and their application in personalized nutrition.

In conclusion, the articles published in this Special Issue will add to the current knowledge in nutrition research using genomics techniques. The increasing knowledge will help nutrition researchers and nutritionists to make more personalized recommendations; thus, it is thought that adherence to dietary advice may increase when it is supported with information based on genomics. Finally, the inclusion of personalized nutrition in our healthcare system may help us to prevent the onset and development of diseases.

## Data Availability

Not applicable.
